# Comparison of the Mortality in Emergency Department Sepsis Score, Modified Early Warning Score, Rapid Emergency Medicine Score and Rapid Acute Physiology Score for predicting the outcomes of adult splenic abscess patients in the emergency department

**DOI:** 10.1371/journal.pone.0187495

**Published:** 2017-11-01

**Authors:** Shang-Kai Hung, Chip-Jin Ng, Chang-Fu Kuo, Zhong Ning Leonard Goh, Lu-Hsiang Huang, Chih-Huang Li, Yi-Ling Chan, Yi-Ming Weng, Joanna Chen-Yeen Seak, Chen-Ken Seak, Chen-June Seak

**Affiliations:** 1 Department of Emergency Medicine, Lin-Kou Medical Center, Chang Gung Memorial Hospital, Taoyuan, Taiwan; 2 College of Medicine, Chang Gung University, Taoyuan, Taiwan; 3 Division of Rheumatology, Allergy and Immunology, Chang Gung Memorial Hospital, Taoyuan, Taiwan; 4 Division of Rheumatology, Orthopedics and Dermatology, School of Medicine, University of Nottingham, Nottingham, United Kingdom; 5 School of Medicine, International Medical University, Kuala Lumpur, Malaysia; 6 Sarawak General Hospital, Kuching, Sarawak, Malaysia; 7 Institute of Occupational Medicine and Industrial Hygiene, College of Public Health, National Taiwan University, Taipei, Taiwan; Azienda Ospedaliero Universitaria Careggi, ITALY

## Abstract

**Background:**

Splenic abscess is rare but has mortality rates as high as 14% even with recent improvements in management. Early and appropriate intervention may improve patient outcomes, yet at present there is no identified method that can predict mortality risk rapidly and accurately for emergency physicians, surgeons, and intensivists to decide on the ideal course of action.

**Objective:**

This study aims to evaluate the performance of Mortality in Emergency Department Sepsis Score (MEDS), Modified Early Warning Score (MEWS), Rapid Emergency Medicine Score (REMS) and Rapid Acute Physiology Score (RAPS) for predicting the mortality risk of adult splenic abscess patients. This will expedite decision making in the emergency department (ED) to increase survival rates and help avoid unnecessary splenectomies.

**Methods:**

Data of 114 adult patients admitted to the EDs of 4 research and training hospitals who had undergone an abdominal contrast CT scan and diagnosed with splenic abscess between Jan 2000 and April 2015 were analyzed. The MEDS, MEWS, REMS, and RAPS and their corresponding mortality risks were calculated, with their abilities to predict patient mortality assessed through receiver operating characteristic curve analysis and calibration analysis.

**Results:**

MEDS was found to be the best performing scoring system across all indicators, with sensitivity, specificity, and accuracy of 92.86%, 88.00%, and 88.60% respectively; its area under curve for AUROC analysis was 0.92. With a cutoff value of 8, negative predictive value of MEDS was 98.88%.

**Conclusion:**

Our series is the largest multicenter study in adult ED patients with splenic abscess. The results from the present study show that MEDS is superior to MEWS, REMS and RAPS in predicting mortality, thus allowing earlier detection of critically ill adult ED splenic abscess patients. Therefore, we recommend that MEDS be used for predicting severity of illness and risk stratification in these patients.

## Introduction

Splenic abscess is a rare but potentially life-threatening disease with an incidence of 0.14% to 0.7% [1–3]. In the past, the mortality rate of patients with splenic abscess was near 100% due to its non-specific presentation and delayed diagnosis [3]. Management of splenic abscess has evolved to now include the improved use of imaging modalities, advanced antibiotic therapy, and timely surgical intervention; even then, the mortality rate still hovers around 14%. The current treatment of choice is intravenous antibiotic therapy with splenectomy, though it exposes patients to the risk of overwhelming post-splenectomy infections. Percutaneous CT-guided drainage is another option, but its superiority over splenectomy has yet to be studied adequately [4]. Because early appropriate intervention may improve the survival outcome of such patients, prompt initiation of the right therapy in the emergency department (ED) after accurate assessment of disease severity and mortality risk is crucial. However, there is still no defined evaluation method that can be promptly and easily used by emergency physicians, surgeons, and intensivists to determine the ideal course of action which effectively utilises medical resources, avoids unnecessary splenectomy, and decreases mortality of these patients.

APACHE II has shown to be a reliable tool to determine the prognosis of patients with splenic abscess [5]. However, given its complexity requiring 14 parameters [6], it does not meet the needs of a rapid risk stratification tool in a regular ED setting. Several ED physiologic scoring systems have been demonstrated as appropriate predictors of the mortality of patients admitted to EDs under different circumstances [7]. Among these, four of them are more commonly used—Rapid Acute Physiology Score (RAPS) [8], Rapid Emergency Medicine Score (REMS) [9–10], Modified Early Warning Score (MEWS) [11], and Mortality in Emergency Department Sepsis Score (MEDS) [12]. These systems share the characteristic of being comprised of simple, rapid, obtainable-by-the-bed parameters that can be calculated immediately, thus allowing for the quick clinical determination of critically-ill patients requiring urgent intervention. To our knowledge, there is yet to be a study to assess the performance of RAPS, REMS, MEWS, and MEDS in the prediction of mortality of ED patients with splenic abscess; hence, this present study was conducted with this purpose.

## Materials and methods

### Study design

This study was a retrospective analysis conducted at the EDs of four training and research hospitals, Linkou Chang Gung Memorial Hospital (3406 beds with approximately 17000 ED visits monthly in 2017), Kaohsiung Chang Gung Memorial Hospital (2686 beds with approximately 12000 ED visits monthly in 2017), Chiayi Chang Gung Memorial Hospital (1375 beds with 5800 ED visits monthly in 2017), and Keelung Chang Gung Memorial Hospital (1089 beds with 5700 ED visits monthly in 2017). The Chang Gung Memorial Hospital Institutional Review Board approved this study (IRB: 201601231B0C501), waiving the need for consent from study participants. Data was accessed anonymously.

### Settings and subjects

We included all adult patients older than 18 years admitted to the EDs of the four hospitals who had undergone an abdominal contrast CT scan in the ED with the final diagnosis of splenic abscess from January 2000 to April 2015.

### Criteria of splenic abscess

Splenic abscess was diagnosed upon meeting any of the following criteria: (1) positive operative findings of splenic abscess during exploratory laparotomy; (2) histologic study of splenic tissue revealed presence of abscess; and if surgery was not performed, (3) presence of clinical manifestations and imaging findings consistent with the diagnosis.

### Measurement of variables

Relevant data was retrieved from the identified patients’ ED medical records, and the physiologic scoring systems (Tables 1–4) were calculated accordingly. *Septic shock* was defined in line with the Second International Consensus Definitions for Sepsis and Septic Shock criteria (2001) [13]. The study endpoint was mortality or survival at the end of hospital stay.

**Table 1:** Rapid Acute Physiology Score (RAPS) scoring system.

	Score				
Variable	0	+1	+2	+3	+4
**PR (/min)**	70–109		55–69 110–139	40–54 140–179	≤39 ≥180
**MAP (mmHg)**	70–109		50–69 110–129	130–159	≤49 ≥160
**RR (/min)**	12–24	10–11 25–34	6–9	35–49	≤5 ≥50
**GCS**	≥14	11–13	8–10	5–7	≤4

PR, pulse rate; MAP, mean arterial pressure; RR, respiratory rate; GCS, Glasgow Coma Scale

**Table 2:** Rapid Emergency Medicine Score (REMS) scoring system.

	Score						
Variable	0	+1	+2	+3	+4	+5	+6
**Age (years)**	<45		45–54	55–64		65–74	>74
**PR (/min)**	70–109		55–69 110–139	40–54 140–179	≤39 >179		
**MAP (mmHg)**	70–109		50–69 110–129	130–159	≤49 >159		
**RR (/min)**	12–24	10–11 25–34	6–9	35–49	≤5 >49		
**GCS**	14 or 15	11–13	8–10	5–7	3 or 4		
**SpO_2_ (%)**	>89	86–89		75–85	<75		

PR, pulse rate; MAP, mean arterial pressure; RR, respiratory rate; GCS, Glasgow Coma Scale; SpO_2_, peripheral oxygen saturation

**Table 3:** Modified Early Warning Score (MEWS) scoring system.

Score				
Variable	0	+1	+2	+3
**Systolic BP (mmHg)**	101–199	81–100	71–80 ≥200	<70
**Heart rate (/min)**	51–100	41–50 101–110	<40 111–129	≥130
**Respiratory rate (/min)**	9–14	15–20	<9 21–29	≥30
**Temperature (°C)**	35–38.4		<35 ≥38.5	
**AVPU score**	**A**lert	Reacts to **V**oice	Reacts to **P**ain	**U**nresponsive

**Table 4:** Mortality in Emergency Department Sepsis (MEDS) scoring system.

Variable	Points
** Terminal illness**^1^	6
** Age > 65 years**	3
** Tachypnea or hypoxia**^2^	3
** Septic shock**	3
** Platelet count < 150 × 10^9^/L**	3
** Band > 5%**	3
** Lower respiratory infection**	2
** Nursing home resident**	2
** Altered mental status**	2

^1^ Defined as rapidly fatal disease with perceived 30-day mortality

^2^ Defined as respiratory rate > 20 breaths/min *or* requiring oxygen by mask *or* SpO_2_ < 90%

### Statistical analysis

Descriptive statistics were presented as median and inter-quartiles for numerical variables and frequencies with their corresponding percentages (%) for categorical variables. Univariate analyses were performed to examine the association between predictors and mortality. Numerical and categorical and variables were analyzed using Mann-Whitney U tests and Fisher’s exact tests respectively, due to small sizes of the non-survivor groups. A logistic regression analysis was performed to develop predictive models between scoring systems and mortality. The probability of death was calculated based on the predictive models using the logit formula:

$$p=\frac{1}{1+\text{exp}\left[-\left(\beta_{0}+\beta_{1}X_{1}\right)\right]}$$

(*β*_0_: Intercept;*β*_1_: Parameter estimate of score; *X*_1_: Score)

Mann-Whitney U tests were also applied to compare the differences in death probabilities between survivors and non-survivors. AUROC analysis was used to compare the predictability of mortality among scoring systems. In addition, the sensitivity, specificity, and accuracy rates were calculated based on the optimal cut-off point derived from the AUROC analysis.

## Results

A total of 114 patients aged 22 years to 84 years (mean 56.33±16.12) were identified in the four hospitals over a span of 15 years and 3 months. The statistically significant results (p < 0.05) are as follows. The pulse rate was 121 versus 106 beats per minute in non-survivors and survivors respectively. Respiratory rate was 22 versus 20 breaths per minute, while mean arterial pressure was 72.5 mmHg versus 94 mmHg in non-survivors and survivors respectively. There was a higher percentage of patients with a poorer Glasgow Coma Scale of <12 in the group of non-survivors at presentation to ED (28.58%) as compared to the group of survivors (2.0%), while platelet count was 162.5 x 10^9^/L versus 221 x 10^9^/L in non-survivors and survivors respectively. The frequency of patients having septic shock was higher in the non-survivor group (42.84%) compared to the survivor group (4.00%) (Table 5).

**Table 5:** Comparison of the characteristics of survivors and non-survivors.

Variable	Patients			
	Total	Survivors	Non-survivors	p-value
No.	114	100	14	
Age (years), Median (IQR)	55.5 (43–72)	55.5 (42–71)	55 (52–78)	0.157
Male, No. (%)	77 (67.54)	70 (70.00)	7 (50.00)	0.221
Body temperature (°C), Median (IQR)	38 (37–39)	38 (36.9–39)	38 (37–39)	0.552
Pulse rate (/min), Median (IQR)*	108 (91–122)	106 (88–117.5)	120.5 (108–142)	0.009
Respiratory rate (/min), Median (IQR)*	20 (19–21)	20 (19–20)	22 (20–27)	0.003
Mean arterial pressure (mmHg), Median (IQR)*	91 (74–111)	94 (75.5–112)	72.5 (59–98)	0.015
Glasgow coma scale, No. (%)*				0.005
≤8	3 (2.63)	1 (1.00)	2 (14.29)	
9–11	3 (2.63)	1 (1.00)	2 (14.29)	
≥12	108 (94.74)	98 (98.00)	10 (71.43)	
Leukocyte count(/μL), Median (IQR)	13000 (8500–18600)	13000 (8950–18550)	13500 (6900–20500)	0.786
Platelets (/μL), Median (IQR)*	217500 (144000–294000)	221000 (152000–303500)	162500 (41000–224000)	0.018
Platelet<150000/μL, No. (%)*	29 (25.44)	22 (22.00)	7 (50.00)	0.044
Septic shock, No. (%)*	10 (8.77)	4 (4.00)	6 (42.84)	<0.001
Terminal illness, No. (%)	2 (1.75)	1 (1.00)	1 (7.14)	0.231
Treatment, No. (%)				0.649
Conservative	58 (50.88)	49 (49.00)	9 (64.29)	
Aspiration	26 (22.81)	24 (24.00)	2 (14.29)	
Operation	30 (26.32)	27 (27.00)	3 (21.43)	
Etiology, No. (%)*				0.025
Hematogenous spread	72 (63.16)	62 (62.00)	10 (71.43)	
Sickle cell disease related	12 (10.53)	8 (8.00)	4 (28.57)	
Traumatic	11 (9.65)	11 (11.00)	0 (0.00)	
Contiguous spread	19 (16.67)	19 (19.00)	0 (0.00)	
Number of abscess, No. (%)*				0.027
Solitary	80 (70.18)	74 (74.00)	6 (42.86)	
Multiple	34 (29.82)	26 (26.00)	8 (57.14)	

* Indicates a statistically significant difference between survivors and nonsurvivors.

Based on the predictive model with the scoring systems using logistic regression analysis, the probability of death was calculated and compared between non-survivors and survivors. The mean probability of death in MEDS was found to be 0.29 in non-survivors and 0.07 in survivors (p < 0.001), while that using MEWS was 0.21 in non-survivors and 0.07 in survivors (p = 0.002). Using RAPS, it was 0.17 in non-survivors and 0.09 in survivors (p = 0.028), and using REMS, it was 0.16 in non-survivors and 0.10 in survivors (p = 0.038) (Table 6).

**Table 6:** The mean and SD of probability of death for the RAPS, MEWS, REMS score, and MEDS values.

Variable	Patients		p-value
	Survivors	Nonsurvivors	
No.	100	14	
MEDS score*	0.07 (0.03–0.07)	0.29 (0.07–0.46)	<0.001
MEWS score*	0.07 (0.03–0.15)	0.21 (0.07–0.37)	0.002
RAPS score*	0.09 (0.05–0.15)	0.17 (0.09–0.25)	0.028
REMS score*	0.10 (0.07–0.14)	0.16 (0.08–0.24)	0.038

* Indicates a statistically significant difference between survivors and non-survivors.

**Fig 1:**
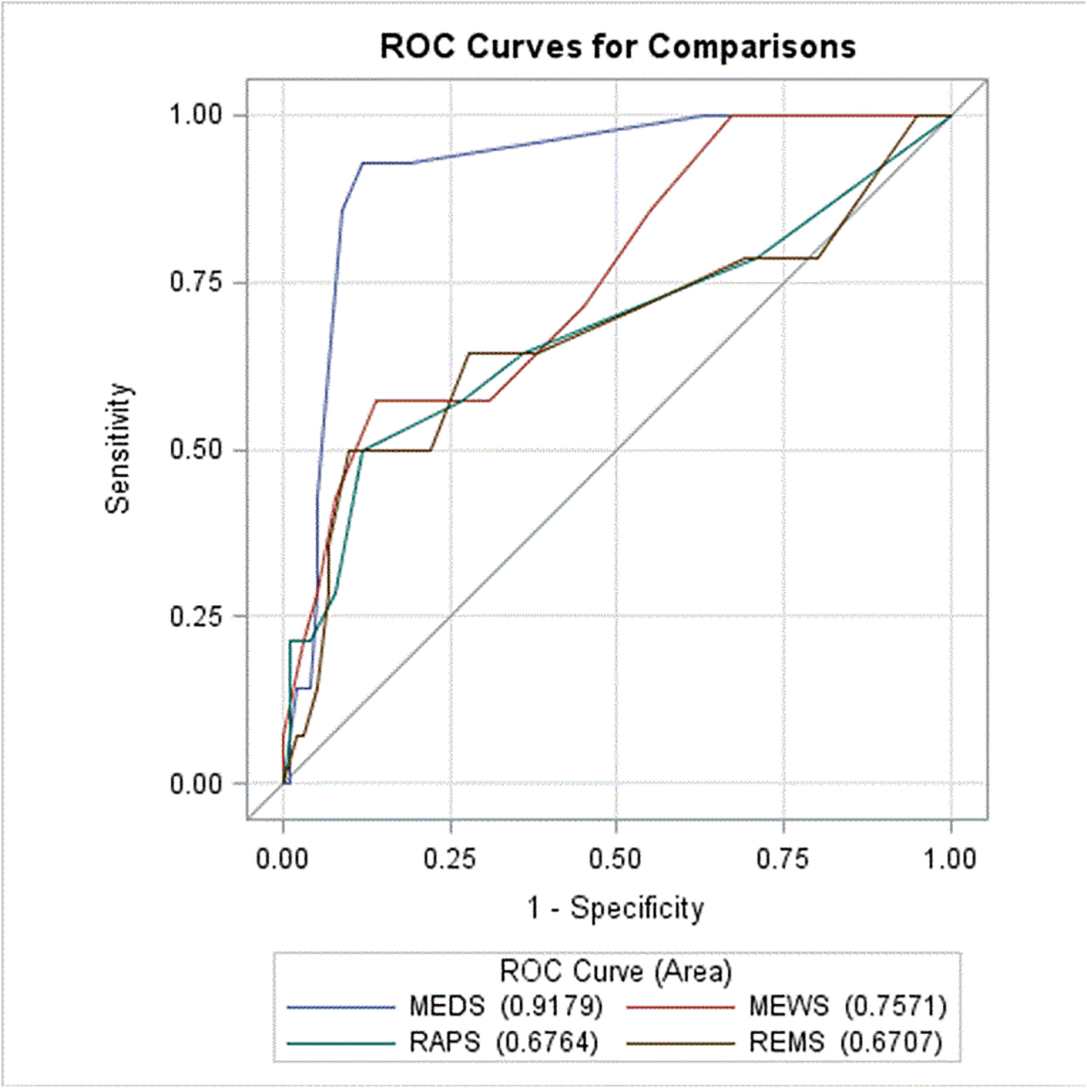
Receiver operating curves for predicting death according the RAPS, MEWS, REMS, and MEDS scoring systems.

The AUROC analysis demonstrated the predictability, in descending order, of MEDS, MEWS, RAPS, and REMS as 0.92, 0.76, 0.68, and 0.67 respectively (Fig 1). MEDS was found to be the most accurate predictive tool, with an accuracy of 88.60%, followed by MEWS at 82.46%. RAPS and REMS ranked the lowest with an accuracy of 71.05%. MEDS was also shown to have the highest sensitivity and specificity amongst the four systems, at 92.86% and 88.00% respectively. With the cutoff value of 8, the negative predictive value of MEDS was found to be 98.88% (Table 7).

**Table 7:** Sensitivities, specificities, and accuracy rates of the RAPS, MEWS, REMS, and MEDS scoring systems for predicting mortality.

Variable	Accuracy rate	Optimal cut-off	Sen	Sp	PPV	NPV
MEDS	88.60%	8	92.86%	88%	52%	98.88%
MEWS	82.46%	6	57.14%	86%	36.36%	93.48%
RAPS	71.05%	4	57.14%	73%	22.86%	92.41%
REMS	71.05%	7	64.29%	72%	24.32%	93.51%

## Discussion

This multi-center study is the largest one to date studying ED patients with the diagnosis of splenic abscess. It is also the first, to our knowledge, that used ED scoring systems for risk assessment and stratification of such patients. In this study, we compared the four scoring systems and found MEDS to be the best performing tool in predicting mortality rates of splenic abscess patients.

MEDS is a prospectively derived and validated clinical prediction rule created by Shapiro et al consisting of 9 parameters, namely age > 65 years, nursing home resident, rapid terminal comorbid illness, lower respiratory tract infection, bands >5% on a WBC differential, tachypnea or hypoxemia, septic shock, platelet count < 150 x 10^9^/L, and altered mental status; the higher the score, the higher the mortality rate. It was designed with the purpose of risk stratification in ED patients with suspected infection according to mortality risk [12]. This is in contrast to the other scoring systems, whose purviews of predicting mortality are not confined solely to patients with infections. This difference in target population might account for the advantage of MEDS over the other 3 systems, as the most common cause of mortality in splenic abscess patients is sepsis and subsequent septic shock. MEDS has also been shown to be a good prognostic indicator in patients with other intra-abdominal infections [14–15]; it has even been found to discriminate better than APACHE II and quick Sepsis-related Organ Failure Assessment in mortality prediction of severe sepsis ED patients [16–18]. Furthermore, in addition to clinical presentation, the MEDS scoring system places emphasis on patient characteristics too– 4 out of 9 criteria (terminal illness, age, nursing home resident, altered mental status) are based on the patient’s medical history, information which can be obtained easily during preliminary history taking in the ED.

Univariate analysis in our study found that pulse rate, respiratory rate, mean arterial pressure, Glasgow Coma Scale, leukocyte count, and presence of septic shock were significant in determining the prognosis of a patient with splenic abscess. Further analysis revealed that thrombocytopenia, commonly found in septic patients [19], was another determinant in our study population. As mentioned earlier, the strong link between septic shock and its predictability of mortality rates is likely to arise from its original designed intentions. The rest of the important parameters are then clinical signs of sepsis: tachycardia, tachypnea, hypotension, altered mental status, and thrombocytopenia. This supports why MEDS score is such a powerful discriminator in determining prognosis of patients with splenic abscess. The high negative predictive value of 98.88% enables emergency physicians, surgeons, and intensivists to quickly screen splenic abscess patients to exclude those with a MEDS score less than 8 from the high mortality risk group; further consideration of the etiology of splenic abscess in our study population showed the same result. These patients may then be conservatively managed without undergoing splenectomy. This holds true even when taking into account that antibiotic therapy and intensive care protocols have evolved dramatically since 2010, as our further analysis revealed that mortality rates did not differ significantly with respect to the patients’ year of hospital admission. While we found etiology of splenic abscess to be a significant determinant of patient mortality upon additional review, it is a major challenge to ascertain this accurately and rapidly in the ED prior to decision making, and therefore is less clinically useful than MEDS in the ED setting.

The AUROC value of MEDS exceeds 0.9, illustrating that it is an excellent tool for predicting splenic abscess mortality. Its sensitivity of 92.86% also allows emergency physicians, surgeons, and intensivists to quickly narrow in with accuracy on the patient requiring urgent intervention. Although MEDS incorporates more parameters as compared to the other 3 scoring systems, the extra information needed are obtainable through history taking and complete blood count investigation commonly done in the evaluation of a patient presenting to the ED.

Despite this study being the largest of its kind, it still has the limitations of being a small study; a larger sample size is required for further confirmation of these findings. Furthermore, this is a retrospective study; future studies can be done prospectively using MEDS score to determine its clinical impact. Due to the fact that this is a study into the most appropriate physiologic scoring system applicable to the ED, we refrained from delving into the specifics of CT imaging interpretation of splenic abscess and its characteristics in deciding treatment; future studies can perhaps address this limitation.

## Conclusion

MEDS score is the best physiologic screening score amongst the four studied scores in predicting mortality of patients with splenic abscess. We recommend using it for rapid risk stratification in the ED, to quickly identify patients requiring urgent intervention, thus ensuring the timeliness of treatment and improving patient outcomes.
